# Evaluation of Cerebral Blood Flow Measured by 3D PCASL as Biomarker of Vascular Cognitive Impairment and Dementia (VCID) in a Cohort of Elderly Latinx Subjects at Risk of Small Vessel Disease

**DOI:** 10.3389/fnins.2021.627627

**Published:** 2021-01-27

**Authors:** Kay Jann, Xingfeng Shao, Samantha J. Ma, Steven Y. Cen, Lina D’Orazio, Giuseppe Barisano, Lirong Yan, Marlena Casey, Jesse Lamas, Adam M. Staffaroni, Joel H. Kramer, John M. Ringman, Danny J. J. Wang

**Affiliations:** ^1^Laboratory of FMRI Technology, USC Mark and Mary Stevens Neuroimaging and Informatics Institute, Keck School of Medicine, University of Southern California, Los Angeles, CA, United States; ^2^Department of Neurology, Keck School of Medicine, University of Southern California, Los Angeles, CA, United States; ^3^Zilkha Neurogenetic Institute and Department of Physiology and Neuroscience, Keck School of Medicine, University of Southern California, Los Angeles, CA, United States; ^4^Department of Neurology, Memory and Aging Center, Weill Institute for Neurosciences, University of California, San Francisco, San Francisco, CA, United States

**Keywords:** arterial spin labeling, item response theory, white matter hyperintensity, cerebral small vessel disease, cerebral blood flow, vascular cognitive impairment and dementia

## Abstract

Cerebral small vessel disease (cSVD) affects arterioles, capillaries, and venules and can lead to cognitive impairments and clinical symptomatology of vascular cognitive impairment and dementia (VCID). VCID symptoms are similar to Alzheimer’s disease (AD) but the neurophysiologic alterations are less well studied, resulting in no established biomarkers. The purpose of this study was to evaluate cerebral blood flow (CBF) measured by 3D pseudo-continuous arterial spin labeling (pCASL) as a potential biomarker of VCID in a cohort of elderly Latinx subjects at risk of cSVD. Forty-five elderly Latinx subjects (12 males, 69 ± 7 years) underwent repeated MRI scans ∼6 weeks apart. CBF was measured using 3D pCASL in the whole brain, white matter and 4 main vascular territories (leptomeningeal anterior, middle, and posterior cerebral artery (leptoACA, leptoMCA, leptoPCA), as well as MCA perforator). The test-retest repeatability of CBF was assessed by intra-class correlation coefficient (ICC) and within-subject coefficient of variation (wsCV). Absolute and relative CBF was correlated with gross cognitive measures and domain specific assessment of executive and memory function, vascular risks, and Fazekas scores and volumes of white matter hyperintensity (WMH). Neurocognitive evaluations were performed using Montreal Cognitive Assessment (MoCA) and neuropsychological test battery in the Uniform Data Set v3 (UDS3). Good to excellent test-retest repeatability was achieved (ICC = 0.77–0.85, wsCV 3–9%) for CBF measurements in the whole brain, white matter, and 4 vascular territories. Relative CBF normalized by global mean CBF in the leptoMCA territory was positively correlated with the executive function composite score, while relative CBF in the leptoMCA and MCA perforator territory was positively correlated with MoCA scores, controlling for age, gender, years of education, and testing language. Relative CBF in WM was negatively correlated with WMH volume and MoCA scores, while relative leptoMCA CBF was positively correlated with WMH volume. Reliable 3D pCASL CBF measurements were achieved in the cohort of elderly Latinx subjects. Relative CBF in the leptomeningeal and perforator MCA territories were the most likely candidate biomarker of VCID. These findings need to be replicated in larger cohorts with greater variability of stages of cSVD.

## Introduction

While Alzheimer’s disease (AD) is the most common cause of dementia, the contribution of vascular factors to cognitive impairment and dementia is becoming increasingly recognized (Gorelick et al., 2011). AD and cerebrovascular diseases share common risk factors such as hypertension, obesity, diabetes, and these conditions coexist in 40–50% of clinically diagnosed AD, making mixed AD-vascular dementia the most common cause of cognitive impairment in the aged (Iadecola, 2016). The clinical differentiation of AD from vascular cognitive impairment and dementia (VCID) is blurred (Schneider et al., 2007). Cerebral small vessel disease (cSVD) is the most common vascular cause of dementia, a major contributor to mixed dementia, and the cause of about one fifth of all strokes worldwide (Norrving, 2008; Pantoni, 2010). The aging population worldwide and the increase in vascular disease with age have led to projections of major growth in VCID over the next 30 years (Gorelick et al., 2011). However, the underlying mechanisms of cSVD remain poorly understood, resulting in no specific guidelines for its prevention and treatment.

The large knowledge gap in cSVD is partly because cerebral small vessels, including arterioles, capillaries, and venules, are inaccessible to existing imaging technologies. Clinical diagnosis of SVD relies on conventional MRI findings including lacunar infarcts, white matter hyperintensities (WMH), cerebral microbleeds, prominent perivascular spaces, and atrophy (Wardlaw et al., 2009). Large epidemiological studies have shown that silent cerebral infarction and WMHs are associated with both non-memory-related cognitive deficits (e.g., executive function and perceptual speed) (Mayda and Decarli, 2009; Rosenberg et al., 2015), and memory impairment (Breteler et al., 1994; DeCarli et al., 1995; Debette et al., 2010). Composite scores of MRI features of cSVD have also been proposed (Staals et al., 2015). However, these parenchymal lesions are the consequences of cSVD rather than the surrogate markers of microvascular changes and cannot guide early interventions to change the course of VCID. It is of paramount importance to identify and develop imaging markers of early microvascular changes related to cSVD for the design of future clinical trials to prevent and treat VCID.

Chronic hypoperfusion is thought to be a key mechanism of SVD and cause of WMHs on MRI. Although the etiology is not completely understood, chronic hypoperfusion may result from the narrowing of the arteriolar lumina secondary to lipohyalinosis and arteriolosclerosis (Yata et al., 2014). A recent systematic review and meta-analysis including 24 cross-sectional studies (*n* = 1161) showed that cerebral blood flow (CBF) is lower in subjects with more WMHs, globally and in most gray and white matter regions (Shi et al., 2016). Arterial spin labeling (ASL) perfusion MRI provides non-invasive quantitative CBF measurement with good test-retest repeatability and has been validated by PET (Kilroy et al., 2014). ASL can be standardized across major MRI platforms with 3D pseudo-continuous ASL (pCASL) (Alsop et al., 2015), which has started to be used in multi-site clinical trials. Therefore, ASL CBF may be applied as a marker of VCID caused by cSVD.

In this study we applied 3D pCASL perfusion MRI to assess the test-retest repeatability of regional CBF for an interval of ∼6 weeks as well as CBF alterations in association with cognitive impairment in a cohort of aged Latinx subjects with varying risks of vascular diseases. Latinx population is the fastest growing segment of the US population who are traditionally underrepresented in clinical research (US Census Bureau, 2016). Latinx population however are at a higher risk for AD and other dementias (Association, 2016), and cerebrovascular disease represents the fourth leading cause of death among Latinx (Heron, 2016). A prominent behavioral phenotype of cSVD is early executive dysfunction manifested by impaired capacity to use complex information, to formulate strategies, and to exercise self-control, with less pronounced episodic memory deficits compared to AD patients (Wallin et al., 2018). Therefore, ASL CBF measurements were correlated with a composite score of executive function developed using Item Response Theory (IRT) (Staffaroni et al., 2020a) and global cognitive measures as well as memory function, vascular risks, and WMHs.

## Materials and Methods

### Human Subjects

Forty-five elderly Latinx subjects (12 males, 69 ± 7 years) participated in the present study as part of the MarkVCID study at the University of Southern California^1^. The inclusion criteria were: (1) Fluency in Spanish and/or English; (2) Age > 60 years; (3) Have capacity for and sign consents indicating so, or give assent and have an appropriate surrogate (as determined by California law) to sign consent; (4) For demented subjects, have an appropriate informant who is also willing and able to accompany the subject. Non-demented subjects must also have an informant willing to participate by phone. The exclusion criteria included history of prior clinical stroke, head trauma, contraindications to MRI, abnormal renal function, pregnancy, other concurrent neurologic or psychiatric illnesses, or abnormal structural MRI (e.g., mass lesions, cystic infarction, etc.). All subjects were required to refrain from caffeine intake and nicotine use 3 h before and during study visits. The demographic and clinical information of the subjects is listed in Table 1.

**TABLE 1:** Demographic characteristics of 45 subjects recruited in this study.

		**Count**	**Column N%**
Sex	Male	12	26.7%
	Female	33	73.3%
Age range (years)	60–92		
Mean ± SD	69 ± 7		
Education (years)	7 ± 4		
Vascular risk factor	Diabetes	17 (of 45)	37.8%
	Hypertension	28 (of 45)	62.2%
	Hypercholesterolemia	33 (of 45)	73.3%
Global CDR scale	0 (Normal)	27	62.8%
	0.5 (Very mild dementia)	16	37.2%
Fazekas scale	0 (No WMH)	6	13.3%
	Periventricular WM		
	1 (Mild WMH)	32	71.1%
	Periventricular WM		
	2 (Severe WMH)	7	15.6%
	Periventricular WM		
	0 (No WMH)	5	11.1%
	Deep WM		
	1 (Mild WMH)	32	71.1%
	Deep WM		
	2 (Severe WMH)	8	17.8%
	Deep WM		

*Vascular risk factors (presence of diabetes, hypertension, or hypercholesterolemia) was defined by a past diagnosis and/or current treatment for these conditions from 45 subjects. Neurocognitive performance was evaluated by global Clinical Dementia Rating (CDR) scale for 43 subjects. Severity of white matter lesions were quantified by the Fazekas scale, which is rated from 0 (absent) to 3 (large confluent areas) in periventricular white matter and deep white matter, respectively.*

### MRI Experiments

MRI scans were performed on a Siemens 3T Prisma system (Erlangen, Germany) using a 20-channel head coil after subjects provided written informed consent according to a protocol approved by the Institutional Review Board (IRB) of the University of Southern California. Each subject underwent repeated MRI scans ∼6 weeks apart (6.7 ± 4.6 weeks). The MRI protocol included a 3D T1-weighted magnetization-prepared rapid gradient-echo (MPRAGE) scan (1 mm^3^ isotropic resolution, TR/TI/TE = 2,530/1,100/1.69 ms), a 3D T2-weighted Fluid-Attenuated Inversion Recovery (FLAIR) scan (1 mm^3^ isotropic resolution, TR/TI/TE = 5,000/1,800/388 ms), and a 3D gradient and spin-echo (GRASE) pCASL scan with background suppression (2.5 mm^3^ isotropic resolution, 48 slices, 4 segments, TR/TE = 4,300 ms/36.8 ms, label duration = 1,500 ms, post-labeling delay = 2,000 ms, 7 label/control image pairs and one M0 image with the same imaging parameters but no background suppression were acquired in 4 min 35 s).

### Clinical and Cognitive Assessments

Clinical evaluation of the participants was performed by a board-certified neurologist (JMR) using the Clinical Dementia Rating (CDR) scores. The CDR is a structured interview of the subject and informant based on which subjects were rated as: 0 (asymptomatic), 0.5 (equivocal impairment), 1 (mild), 2 (moderate), or 3 (severe dementia). All participants were rated CDR 0 or 0.5 (Table 1). The Clinical Dementia Rating Sum of Boxes (CDR-SOB) scores were used for correlation analyses with CBF due to the wider score range of 0–18 (O’Bryant et al., 2008). The Montreal Cognitive Assessment (MoCA) was performed on 39 participants by a licensed neuropsychologist (LD) with scores ranging from 0 to 30 (Nasreddine et al., 2005). Testing was administered in English (5, 13%) or Spanish (34, 87%), determined by the comfort level of the subjects. Neurocognitive evaluations were performed using standard neuropsychological test battery in the Uniform Data Set v3 (UDS3). Domain specific assessment of executive function was performed using scores of Trail Making B—A and a composite score of executive function developed using IRT (Staffaroni et al., 2020a). Composite scores of executive function offer advantages such as better reliability, fewer statistical comparisons, and improved power to detect longitudinal change with smaller sample sizes (Gibbons et al., 2012; Staffaroni et al., 2020b). Memory scores were derived from the Spanish English Verbal Learning Test (SEVLT) which has been shown to be appropriate and have adequate normative data for use with older Latinx persons of Mexican descent (González et al., 2002). The memory scores included Total Learned (over the learning trials), Benefit of Cues (difference between delayed free recall and delayed cued recall), and percentage retained (Delayed Free Recall / Trial 5). Overall, 43 subjects had complete behavioral and clinical assessments.

Furthermore, presence or absence of hypertension, diabetes, and hypercholesterolemia (0 or 1) was defined by a past diagnosis and/or current treatment for these conditions. Vascular risk factor (0–3) was calculated as the combination of presences of hypertension, diabetes, or hypercholesterolemia, which was used for correlation analysis with CBF.

### Data Analysis

ASL data were preprocessed and quantified using SPM12 and in-house Matlab scripts. Following motion correction, pair-wise subtraction of label and control images was performed and averaged across the time series to generate mean perfusion images. Quality control metrics were generated by the program and included framewise displacement for head motion and temporal SNR. Quantitative CBF maps were generated using the standard one-compartment perfusion model (Alsop et al., 2015) using voxel-wise calibration with the M0 image. Results were visually checked for any artifacts. Only one CBF dataset was found to have artifacts with abnormally low CBF, and this subject (66 years, F) was excluded for further analyses. CBF maps from the 1st and 2nd visit of the remaining 44 subjects were coregistered to individual T1w MRI and normalized to the Montreal Neurological Institute (MNI) template space. CBF was measured in the whole brain (WB), white matter (WM) and 4 main vascular territories [leptomeningeal anterior cerebral artery (ACA), middle cerebral artery (MCA) and posterior cerebral artery (PCA), as well as MCA perforator] using a template (Tatu et al., 1998; Wang et al., 2012). WM mask was created from tissue probability maps thresholded at 99%.

White matter hyperintensity (WMH) was segmented from T2-weighted FLAIR images using ITK-SNAP^2^ (Yushkevich et al., 2006) and its semi-automatic segmentation tool for supervised classification based on random forests. Three tissue classes (WMH, non-hyperintense tissue, and CSF) were identified as training examples for the classifier, and seeds were then placed in the observed WMH. The active contour algorithm was used to iteratively expand the seeds into the WMH segmentation, which was then carefully inspected by clinical fellows and manually adjusted if necessary. The final segmentation was used to quantify the total WMH volume which was normalized by the intracranial volume (ICV) segmented and measured using SPM12 (FIL, UCL, London, United Kingdom) in each subject. Severity of white matter lesions were quantified by the Fazekas scale (Fazekas et al., 1987), which is rated from 0 (absent) to 3 (large confluent areas) in periventricular white matter and deep white matter, respectively. Since Fazekas scores of periventricular and deep WM were highly correlated, we used total Fazekas scores for correlation analysis with CBF.

The test-retest repeatability of CBF was assessed by intra-class correlation coefficient (ICC) and within-subject coefficient of variation (wsCV). Absolute and relative CBF (vs. global mean CBF) were correlated with global cognitive measures, CDR-SOB scores and domain specific assessment of executive and memory function, with age, gender, years of education and testing language as covariates. Absolute and relative CBF (normalized by global mean CBF) were also correlated with vascular risk factor, Fazekas scores, and volumes of WMH, with covariates of age and gender. Data distribution was examined using histogram and Shapiro–Wilk test which has relatively strong statistical power to be suitable for small sample size, compared to other normality tests. Logarithm transformation was applied to skewed data when necessary. For unadjusted correlation, either Pearson or Spearman correlation was used depending on normality. To examine the adjusted correlation, data were first rescaled to z scores then fit with generalized linear multivariate regression model (GLM) with covariates. The beta coefficient from the regression model using z score can approximate the correlation coefficient and be comparable between variables of interest. For Fazekas scores, since they are categorical measurements (k levels), GLM with k-1 degree of freedom ANOVA test was used as the global association test. Residual plots were used to assess the model integrity and Cook’s d was used to detect outliers with extreme influence. Multivariate Adaptive Regression Spline (MARS) (Friedman, 1991) was used to explore the potential non-linear association. Scatter plots were used to illustrate the correlation with outliers highlighted. If a spline detected by MARS, the spline regression slope instead of linear regression slope was used to illustrate the potential non-linear association. Based on our hypothesis and literature evidence, we focused on CBF measurements in 3 regions for correlation with behavioral and imaging biomarkers of cSVD: leptomeningeal MCA (leptoMCA) and MCA perforator (MCAperf) territories, and WM. As an exploratory analysis, an α level of 0.05 (2-sided) was used for statistical significance without correction for multiple comparisons. SAS9.4 was used for statistical analysis.

## Results

### Test-Retest Repeatability of CBF

**FIGURE 1:**
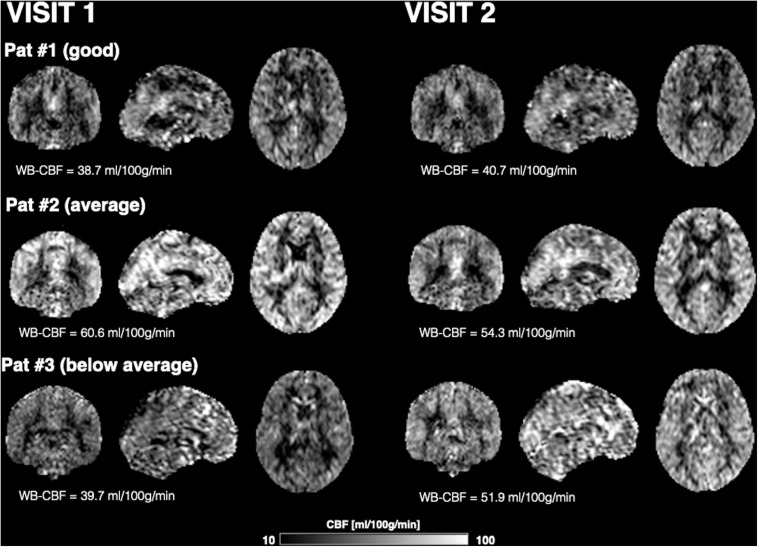
CBF maps normalized to the MNI canonical space and displayed in coronal, sagittal, and axial views of three representative participants acquired on two visits. The three participants had above average, average and below average reproducibility of repeated CBF measurements.

**FIGURE 2:**
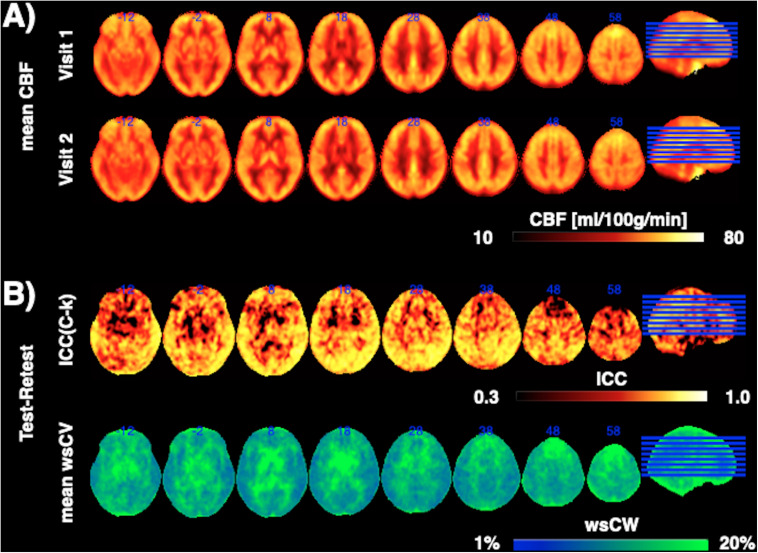
**(A)** Mean CBF maps for visit1 and visit2 as well as **(B)** the voxel-wise ICC and wsCW maps of test-retest results for all the 44 subjects.

**FIGURE 3:**
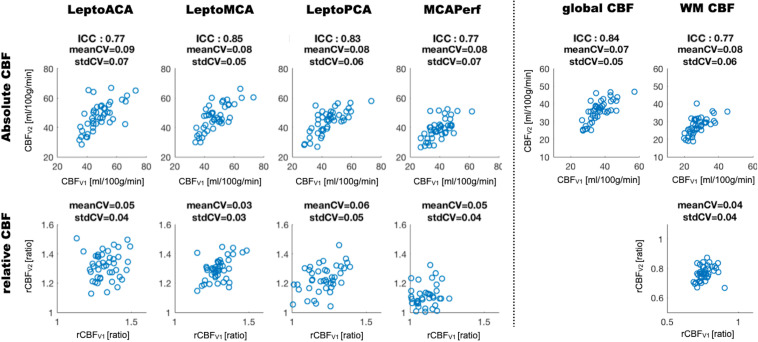
Scatter plots of absolute and relative CBF values acquired on two visits measured in the whole brain (global CBF), white matter (WM) and 4 vascular territories. ICC and wsCV (mean and SD) of each ROI are listed for absolute CBF while wsCV values are listed for relative CBF.

Figure 1 shows CBF maps normalized to the MNI canonical space of three representative participants acquired on two visits. The three participants had above average, average and below average reproducibility of repeated CBF measurements, respectively. Mean CBF maps for visit1 and visit2 as well as the voxel-wise ICC and wsCW maps of test-retest results for all the 44 subjects are shown in Figure 2. Good to excellent test-retest repeatability was achieved (ICC = 0.77–0.85, wsCV 3–9%, Figure 3) for absolute CBF measurements in the WB [ICC(C-k) = 0.84], WM [ICC(C-k) = 0.77], and 4 vascular territories: leptomeningeal ACA (leptoACA, ICC_abs_ = 0.77, wsCV_abs_ = 0.09 ± 0.07); leptomeningeal MCA (leptoMCA, ICC_abs_ = 0.85, wsCV_abs_ = 0.08 ± 0.05); leptomeningeal PCA (leptoPCA, ICC_abs_ = 0.83, wsCV_abs_ = 0.08 ± 0.06);, and MCA perforator territory (MCAperf, ICC_abs_ = 0.77, wsCV_abs_ = 0.08 ± 0.07). For relative CBF, wsCV was calculated for test-retest repeatability since the inter-subject variation was very small due to normalization by global CBF. The wsCV of relative CBF ranged from 3 to 6% in the 4 vascular territories between repeated scans ∼6 weeks apart (Figure 3): leptoACA (wsCV_rel_ = 0.05 ± 0.04); leptoMCA (wsCV_rel_ = 0.03 ± 0.03); leptoPCA (wsCV_rel_ = 0.06 ± 0.05);, and MCAperf (wsCV_rel_ = 0.05 ± 0.04).

### Correlations Between CBF and Cognitive Scores

**FIGURE 4:**
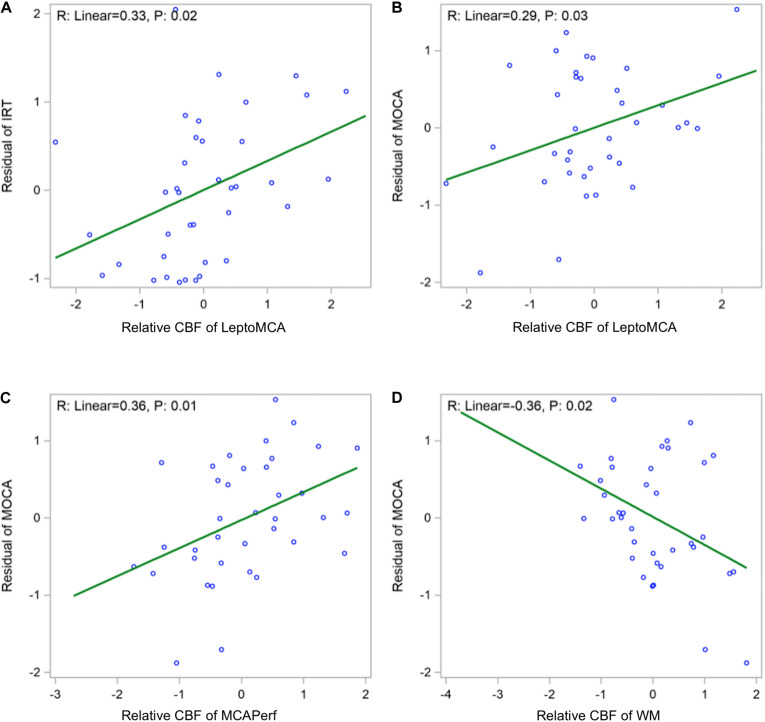
Scatter plots showing **(A)** positive correlations between relative CBF in the leptoMCA and executive function composite score [adjusted beta = 0.33, 95% CI (0.08, 0.59), *P* = 0.02]; **(B)** positive correlation between relative CBF in the leptoMCA territory and MoCA score [β = 0.29, 95% CI (0.04, 0.54), *P* = 0.03]; **(C)** positive correlation between relative CBF in the MCAperf territory and MoCA score [β = 0.36, 95% CI (0.10, 0.63), *P* = 0.01]; **(D)** negative correlation between relative CBF in WM and MoCA scores [β = –0.36, 95% CI (–0.66, –0.07), *P* = 0.02). Final associations were controlled for age, gender, years of education and testing language, and used standardized z scores for both independent and dependent variables.

Global mean CBF was not significantly correlated with any cognitive scores. Relative CBF normalized by global mean CBF in the leptoMCA territory was positively correlated with the executive function composite score [Figure 4A, beta = 0.33, 95% CI (0.08, 0.59), *P* = 0.02]. Relative CBF in the MCAperf territory [Figure 4B, beta = 0.29, 95% CI (0.04, 0.54), *P* = 0.03] and leptoMCA territory [Figure 4C, beta = 0.36, 95% CI (0.10, 0.63), *P* = 0.01] was positively correlated with MoCA scores, controlling for age, gender, years of education and testing language. However, relative CBF in WM was negatively correlated with MoCA scores [Figure 4D, beta = −0.36, 95% CI (−0.66, −0.07), *P* = 0.02], controlling for age, gender, years of education and testing language.

For association with memory function, no CBF measurements in leptoMCA or MCAperf territories were found to be correlated with any of the three SEVLT scores. However, relative CBF of WM was positively correlated with the Benefit of Cues score of SEVLT [beta = 0.5, 95% CI (0.09, 0.9), *P* = 0.022]. No CBF measurements were significantly correlated with CDR-SOB scores.

### Correlations Between CBF, Vascular Risks and WMH

No CBF measurements were found to be correlated with vascular risk factor—the combination of presences of hypertension, diabetes, or hypercholesterolemia (0–3).

**FIGURE 5:**
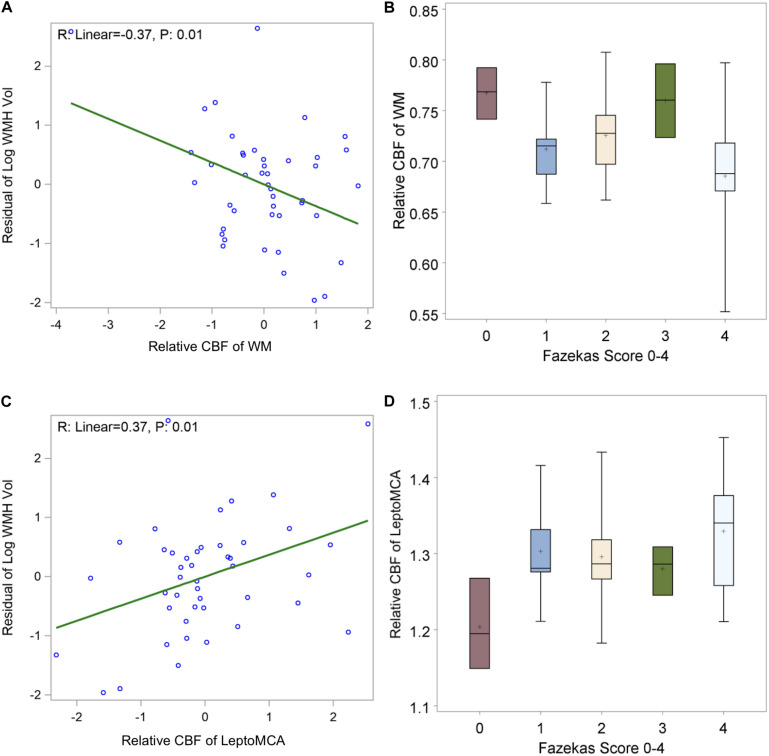
shows **(A)** negative correlations between relative WM CBF and log transformation of normalized WMH volume [β = –0.37, 95% CI (–0.64, –0.10), *P* = 0.01 adjusted for age and gender]; **(B)** linear decreasing trend of relative WM CBF by increasing in total Fazekas scores of WMH [β = –0.02, 95% CI (–0.0004, –0.04) *P*: 0.02]; **(C)** positive correlations between relative leptoMCA CBF with the log transformation of normalized WMH volume [β = 0.37, 95% CI (0.10, 0.64), *P* = 0.01 adjusted for age and gender]; **(D)** linear increasing trend of relative leptoMCA CBF by increasing in total Fazekas scores of WMH [β = 0.03, 95% CI (0.01, 0.05) *P*: < 0.01].

The WMH was found to be generally mild (WMH volume < 20 ml) in the study cohort except two subjects with WMH volume greater than 60 ml (top two dots in Figure 5A). We found a significant negative correlation between relative WM CBF and the log transformation of normalized WMH volume [Figure 5A, beta = −0.37, 95% CI (−0.64, −0.10), *P* = 0.01], controlling for age and gender. There was also a negative correlation between relative WM CBF and total Fazekas scores of WMH (Figure 5B, *p* = 0.03). However, relative leptoMCA CBF was positively correlated with the log transformation of normalized WMH volume [Figure 5C, beta = 0.37, 95% CI (0.10, 0.64), *P* = 0.01] and total Fazekas scores of WMH (Figure 5D, *p* = 0.02).

## Discussion

In this study, we evaluated CBF measurement by 3D pCASL as a potential biomarker of VCID in a cohort of elderly Latinx subjects at risk of cSVD. Good to excellent test-retest repeatability was achieved (ICC = 0.77–0.85, wsCV 3–9%) for CBF measurements in the whole brain, WM, and 4 vascular territories. Relative CBF normalized by global mean CBF in the leptoMCA territory was positively correlated with an IRT-derived composite score of executive function, while relative CBF in the MCA perforator and leptoMCA territory was positively correlated with MoCA scores of global cognitive function. Relative CBF in WM was negatively correlated with WMH volume and MoCA scores, while relative leptoMCA CBF was positively correlated with WMH volume.

### Test-Retest Repeatability of CBF

The test-retest repeatability of ASL CBF measurements has been evaluated using different ASL techniques in different populations. Chen et al. (2011) compared three common ASL strategies with 2D EPI readout: pCASL, pulsed (PASL) and continuous ASL (CASL) in 12 young healthy subjects at 3T, with scan intervals up to 1 week. The authors reported a wsCV on the order of 10% for GM CBF for PASL (9.2 ± 0.12%) and pCASL (8.5 ± 0.14%), with higher variability for longer scan intervals. Kilroy et al. (2014) evaluated repeatability (∼4 weeks apart) and accuracy (by comparing to 15O-PET) of pCASL with 2D EPI and 3D GRASE readout in 13 elderly subjects including six MCI and one mild AD. GRASE pCASL demonstrated a higher repeatability for regional perfusion measurements (ICC = 0.707, wsCV = 10.9%) compared to EPI pCASL (ICC = 0.362, wsCV = 15.3%). Hodkinson et al. (2013) evaluated 3D pCASL in 16 healthy male volunteers (age range: 18–50 years) in a clinical model of post-surgical pain. The inter- and intra-session reliability of the post-surgical pCASL CBF measurements were good-to-excellent (ICC > 0.6), while the repeatability of ΔCBF between pre- and post-surgical states was moderate (ICC > 0.4). Between-subjects, the pCASL CBF measurements in the post-surgical pain state and ΔCBF were both characterized as moderately reliable (ICC > 0.4) across nine ROIs. Lin et al. (2020) compared test-retest repeatability of 3D pCASL with standard (1,500 ms) and long (3,500 ms) labeling duration in 20 adult volunteers (age 56.6 ± 17.2 years) with a 1 h interval. The ICCs were generally high (> 0.85) and wsCV < 10% across lobar ROIs with the long labeling duration pCASL showing higher repeatability.

Overall our results of test-retest repeatability of 3D pCASL CBF measurements in the cohort of elderly Latinx subjects are consistent with those reported in literature. To the best of our knowledge, this is the first pCASL study in Latinx population with a moderate sample size (*N* = 45 recruited from communities) that is larger than those in previous studies. The scan interval of ∼6 weeks is also longer than those in previous studies, adding challenges for achieving a high repeatability. Nevertheless, the test-retest repeatability of 3D pCASL CBF measurements in our study was comparable and even higher than those reported in literature. Our data strongly support that 3D pCASL CBF measurement can be applied as an imaging biomarker in community based clinical studies/trials.

### Correlation of CBF With Neurocognitive Function

In this study, relative CBF in the leptoMCA territory was correlated with executive dysfunction (Staffaroni et al., 2020b) which is considered a prominent behavioral phenotype of cSVD. In contrast, episodic memory deficits are more pronounced in patients with AD (Wallin et al., 2018). The leptoMCA territory includes a large area of cerebral cortex including the frontal, parietal and temporal lobes. These regions comprise the default mode, dorsal attention and frontoparietal networks associated with attention and executive functions (22). A recent study also found positive associations between the executive function composite score and CBF in dorsolateral prefrontal cortex (Staffaroni et al., 2019). Therefore, it is not surprising that relative CBF in leptoMCA territory was correlated with the executive function composite score. In addition, leptoMCA relative CBF was correlated with MoCA scores of gross cognition. This finding is not surprising given the involvement of leptoMCA territory in a wide range of cognitive functions. A previous study in a cohort of Chinese patients with subcortical vascular cognitive impairment found diffuse global reductions in CBF and correlation of regional CBF with gross cognition in temporal, orbitofrontal and insular cortices (Sun et al., 2016). These areas partially overlap with the areas of the leptoMCA territory. A main difference is that the previous study did not account for the widespread global hypoperfusion, a factor that will be discussed below.

We also observed that relative CBF in the MCA perforator territory was positively correlated with MoCA scores of global cognitive function. The MCA perforator territory primarily consists of lenticulostriate arteries (LSAs) that supply important subcortical areas including the caudate nucleus, globus pallidus, putamen, and part of the posterior limb of the internal capsule (Ma et al., 2019). LSAs take origin directly from the high flow MCA with small diameters on the order of a few hundred microns (Marinković et al., 2001), therefore are particularly susceptible to damage (e.g., by hypertension) (Dichgans and Leys, 2017). Indeed, subcortical ischemic infarcts (e.g., lacunes) are a prominent hallmark of cSVD (Wardlaw et al., 2009). In addition, CBF in leptoMCA or MCA perforator territories were not associated with any of the 3 memory scores derived from the SEVLT. Relative MCAperf CBF was also correlated with retinal capillary density measured by optical coherence tomography angiography (OCTA) in the same cohort of Latinx subjects (Ashimatey et al., 2020). Our findings, in conjunction with existing evidence, prompt relative CBF in the leptomeningeal and perforator MCA territories as candidate biomarkers of VCID. However, the area of MCA perforator territory is much smaller than that of leptoMCA which may affect the reliability of CBF measurements (as indicated by lower ICC values in the former).

Previous studies have shown the advantages of using psychometrically robust composite scores over a single or multiple cognitive test scores to quantify cognitive performance (Crane et al., 2012; Gibbons et al., 2012; Staffaroni et al., 2020b). The IRT-based score used in the present study is sensitive to impairments in MCI, AD, and frontotemporal dementia (Staffaroni et al., 2020b). Furthermore, it was associated with gray matter volume of frontal, parietal, and temporal lobes, the same regions that make up the leptoMCA territory. A different IRT-based composite score of executive function derived from the NIH-EXAMINER has been shown to detect longitudinal declines in asymptomatic carriers of mutations that cause frontotemporal dementia and is sensitive to premanifest Huntington’s disease (You et al., 2014), two conditions that present with executive dysfunction. This suggests these composite scores may be sensitive to the earliest changes in executive abilities. In the present study, we did not observe associations between CBF and another common metric of executive function (Trail Making B—A), providing additional evidence that an IRT-based composite score may provide a more robust measure of executive function than its component tests.

We found relative CBF in WM was negatively correlated with MoCA scores, which contradicts the positive association between MCA CBF and MoCA scores. The reason for this observation is not known. It is possible that normalization with global mean CBF may play a role in different trends of relative CBF between GM and WM regions. However, absolute CBF was not associated with behavioral measures or WMH in our study, suggesting the importance of controlling variations in global CBF with normalization for regional CBF values. This notion is in line with previous reports that inter-individual differences in global CBF are higher than within-subject CBF variations (Henriksen et al., 2012), and thus, detection of small inter-subject regional CBF variations associated with cognitive or clinical assessments requires global CBF bias correction or normalization. It also has been demonstrated that relative CBF is more sensitive in detecting regional differences across subjects compared to absolute CBF (Aslan and Lu, 2010).

### Correlation of CBF With WMH

WMH is the most prominent imaging feature of cSVD (Wardlaw et al., 2009). WMHs are associated with increased risk of cognitive impairment and dementia (Alber et al., 2019), and are presumed to be caused by chronic hypoperfusion, blood-brain barrier (BBB) breakdown, dysfunction of oligodendrocyte precursor cells, and venous collagenosis (Kim et al., 2020). A recent systematic review and meta-analysis with a large pooled sample (*n* = 1,161) showed that CBF is lower in subjects with more WMHs (Shi et al., 2016). Several studies also investigated perfusion changes in the WMH penumbra, which is the normal appearing WM around the hyperintensities (Promjunyakul et al., 2015, 2016; Rane et al., 2018). These studies found that hypoperfusion in the WMH penumbra are associated with cognition and future WMH growth. In the present study, we found a significant negative correlation between relative WM CBF and the log transformation of normalized WMH volume, as well as total Fazekas scores of WMH. This finding is consistent with the hypothesis of chronic hypoperfusion as a main etiology of WMH in literature. However, this trend was reversed for relative CBF in leptoMCA which is positively correlated with the log transformation of normalized WMH volume and total Fazekas scores of WMH. The WMH was generally mild (WMH volume < 20 ml) in the study cohort except two subjects with WMH volume greater than 60 ml (top two dots in Figure 5A). Excluding these two subjects with large WMH volume, none of the reported associations between CBF and WMH were significant (*P* > 0.05). Therefore, the reported findings need to be interpreted with caution and should be verified in larger cohorts with greater variability of WMH.

### Limitations and Future Directions

There are several limitations of this study: (1) The sample size is moderate and the study cohort is all aged Latinx subjects. This is both a strength and weakness since our study is the first of its kind in a Latinx population; nevertheless, the relatively uniform cohort may not have large enough variability in the stages of cSVD (e.g., WMH); (2) Our study was performed on a single 3T MRI scanner. It would be ideal to evaluate the test-retest repeatability of ASL CBF across MRI scanners by different manufacturers, as shown by Mutsaerts et al. (2015). This has become feasible with the recommended implementation of pCASL with background suppressed 3D acquisitions across major MRI platforms (Alsop et al., 2015); (3) We did not perform correction for multiple comparisons for exploratory analyses. The sample size was only powered for reliability test, but was underpowered for testing the association with clinical outcomes. Such an exploratory analysis can provide us the insight for directing further confirmative studies with larger sample sizes.

## Conclusion

Reliable 3D pCASL CBF measurements were achieved in the cohort of elderly Latinx subjects. Relative CBF in the leptomeningeal and perforator MCA territories were the most likely candidate biomarker of VCID. These findings need to be replicated in larger cohorts with greater variability of stages of cSVD.

## Data Availability Statement

The datasets presented in this article was collected as part of the MarkVCID consortium study at the University of Southern California (www.markvcid.org). The datasets is available by contacting the corresponding author.

## Ethics Statement

The studies involving human participants were reviewed and approved by the Institutional Review Board of the University of Southern California. The patients/participants provided their written informed consent to participate in this study.

## Author Contributions

KJ, XS, SM, and DW developed the post processing pipeline, conducted data analysis, and wrote the manuscript. XS, KJ, SM, LY, MC, JR, LD’O, and DW carried out the experiments and collected data. SC, KJ, SM, GB, AS, JK, and JL conducted data analysis. LD’O, JL, KJ, LY, AS, JR, and DW guided experiments, discussed results, and revised manuscript. All authors edited and revised the manuscript and approved final submission.

## Conflict of Interest

The authors declare that the research was conducted in the absence of any commercial or financial relationships that could be construed as a potential conflict of interest.

## Footnotes

1 www.markvcid.org

2 www.itksnap.org

## References

[B1] Alber J. Alladi S. Bae H.J. Barton D.A. Beckett L.A. Bell J.M. (2019). White matter hyperintensities in vascular contributions to cognitive impairment and dementia (VCID): Knowledge gaps and opportunities. *Alzheimers Dement* 5 107–117. 10.1016/j.trci.2019.02.001 PMC6461571 31011621

[B2] Alsop D.C. Detre J.A. Golay X. Gunther M. Hendrikse J. Hernandez-Garcia L. (2015). Recommended implementation of arterial spin-labeled perfusion MRI for clinical applications: a consensus of the ISMRM perfusion study group and the european consortium for ASL in dementia. *Magn. Reson. Med.* 73 102–116. 10.1002/mrm.25197 24715426 PMC4190138

[B3] Ashimatey B.S. D’Orazio L.M. Ma S.J. Jann K. Jiang X. Lu H. (2020). Lower retinal capillary density in minimal cognitive impairment among older latinx adults. *Alzheimers Dement.* 12:e12071. 10.1002/dad2.12071 PMC7447879 32875053

[B4] Aslan S. Lu H. (2010). On the sensitivity of ASL MRI in detecting regional differences in cerebral blood flow. *Magn. Reson Imaging* 28 928–935. 10.1016/j.mri.2010.03.037 20423754 PMC2912434

[B5] Association A.S. (2016). 2016 Alzheimer’s disease facts and figures. *Alzheimers Dement.* 12 459–509. 10.1016/j.jalz.2016.03.001 27570871

[B6] Breteler M.M. van Amerongen N.M. van Swieten J.C. Claus J.J. Grobbee D.E. van Gijn J. (1994). Cognitive correlates of ventricular enlargement and cerebral white matter lesions on magnetic resonance imaging. The rotterdam study. Stroke 25 1109–1115. 10.1161/01.str.25.6.1109 8202966

[B7] Chen Y. Wang D.J.J. Detre J.A. (2011). Test-retest reliability of arterial spin labeling with common labeling strategies. *J. Magn. Reson. Imaging* 33 940–949. 10.1002/jmri.22345 21448961 PMC3069716

[B8] Crane P.K. Carle A. Gibbons L.E. Insel P. Mackin R.S. Gross A. (2012). Development and assessment of a composite score for memory in the Alzheimer’s Disease neuroimaging Initiative (ADNI). *Brain Imaging Behav.* 6 502–516. 10.1007/s11682-012-9186-z 22782295 PMC3806057

[B9] Debette S. Beiser A. DeCarli C. Au R. Himali J.J. Kelly-Hayes M. (2010). Association of MRI markers of vascular brain injury with incident stroke, mild cognitive impairment, dementia, and mortality: the framingham offspring study. Stroke 41 600–606. 10.1161/strokeaha.109.570044 20167919 PMC2847685

[B10] DeCarli C. Murphy D.G. Tranh M. Grady C.L. Haxby J.V. Gillette J.A. (1995). The effect of white matter hyperintensity volume on brain structure, cognitive performance, and cerebral metabolism of glucose in 51 healthy adults. Neurology 45 2077–2084. 10.1212/wnl.45.11.2077 7501162

[B11] Dichgans M. Leys D. (2017). Vascular cognitive impairment. *Circ. Res.* 120 573–591. 28154105 10.1161/CIRCRESAHA.116.308426

[B12] Fazekas F. Chawluk J.B. Alavi A. Hurtig H.I. Zimmerman R.A. (1987). MR signal abnormalities at 1.5 T in Alzheimer’s dementia and normal aging. *Am. J. Roentgenol.* 149 351–356. 10.2214/ajr.149.2.351 3496763

[B13] Friedman J.H. (1991). Multivariate adaptive regression splines. *Annals Stat.* 19 1–67.

[B14] Gibbons L.E. Carle A.C. Mackin R.S. Harvey D. Mukherjee S. Insel P. (2012). A composite score for executive functioning, validated in Alzheimer’s disease neuroimaging initiative (ADNI) participants with baseline mild cognitive impairment. *Brain Imaging Behav.* 6 517–527. 10.1007/s11682-012-9176-1 22644789 PMC3684181

[B15] González H.M. Mungas D. Haan M.N. (2002). A verbal learning and memory test for english- and spanish-speaking older mexican-american adults. *Clin. Neuropsychol.* 16 439–451. 10.1076/clin.16.4.439.13908 12822053

[B16] Gorelick P.B. Scuteri A. Black S.E. Decarli C. Greenberg S.M. Iadecola C. (2011). Vascular contributions to cognitive impairment and dementia: a statement for healthcare professionals from the american heart association/american stroke association. *Stroke* 42 2672–2713. 10.1161/str.0b013e3182299496 21778438 PMC3778669

[B17] Henriksen O.M. Larsson H.B. Hansen A.E. Grüner J.M. Law I. Rostrup E. (2012). Estimation of intersubject variability of cerebral blood flow measurements using MRI and positron emission tomography. *J. Magn. Reson. Imaging* 35 1290–1299. 10.1002/jmri.23579 22246715

[B18] Heron M., (2016). Deaths: leading causes for 2014. *Natl. Vital. Stat. Rep.* 65 1–96. 27376998

[B19] Hodkinson D.J. Krause K. Khawaja N. Renton T.F. Huggins J.P. Vennart W. (2013). Quantifying the test-retest reliability of cerebral blood flow measurements in a clinical model of on-going post-surgical pain: a study using pseudo-continuous arterial spin labelling. *Neuroimage Clin.* 3 301–310. 10.1016/j.nicl.2013.09.004 24143296 PMC3797555

[B20] Iadecola C. (2016). Vascular and metabolic factors in alzheimer’s disease and related dementias: introduction. *Cell Mol. Neurobiol.* 36 151–154 10.1007/s10571-015-0319-y 26898551 PMC4846525

[B21] Kilroy E. Apostolova L. Liu C. Yan L. Ringman J. Wang D.J. (2014). Reliability of two-dimensional and three-dimensional pseudo-continuous arterial spin labeling perfusion MRI in elderly populations: comparison with 15O-water positron emission tomography. *J. Magn.Reson. Imaging* 39 931–939. 10.1002/jmri.24246 24038544 PMC3866214

[B22] Kim H.W. Hong J. Jeon J.C. (2020). Cerebral small vessel disease and alzheimer’s disease: a review. *Front. Neurol.* 11:927. 10.3389/fneur.2020.00927 32982937 PMC7477392

[B23] Lin T. Qu J. Zuo Z. Fan X. You H. Feng F. (2020). Test-retest reliability and reproducibility of long-label pseudo-continuous arterial spin labeling. *Magn Reson Imaging* 73 111–117. 10.1016/j.mri.2020.07.010 32717203

[B24] Ma S.J. Sarabi M.S. Yan L. Shao X. Chen Y. Yang Q. (2019). Characterization of lenticulostriate arteries with high resolution black-blood T1-weighted turbo spin echo with variable flip angles at 3 and 7 tesla. *Neuroimage* 199 184–193. 10.1016/j.neuroimage.2019.05.065 31158475 PMC6688958

[B25] Marinković S. Gibo H. Milisavljević M. Ćetković M. (2001). Anatomic and clinical correlations of the lenticulostriate arteries. *Clin. Anatomy* 14 190–195. 10.1002/ca.1032 11301466

[B26] Mayda A. Decarli C. (2009). Vascular cognitive impairment: prodrome to VaD? In:. In: Wahlund L-O Erkinjuntti T. Gauthier S (Eds.). *Vascular Cognitive Impairment in Clinical Practice.* Cambridge,. Cambridge University Press 11–31. 10.1017/cbo9780511575976.003

[B27] Mutsaerts H.J.M.M. van Osch M.J.P. Zelaya F.O. Wang D.J.J. Nordhoy W. Wang Y. (2015). Multi-vendor reliability of arterial spin labeling perfusion MRI using a near-identical sequence: implications for multi-center studies. *Neuroimage* 113 143–152. 10.1016/j.neuroimage.2015.03.043 25818685

[B28] Nasreddine Z.S. Phillips N.A. Bédirian V. Charbonneau S. Whitehead V. Collin I. (2005). The montreal cognitive assessment, MoCA: a brief screening tool for mild cognitive impairment. *J. Am. Geriat. Soc.* 53 695–699. 15817019 10.1111/j.1532-5415.2005.53221.x

[B29] Norrving B. (2008). Lacunar infarcts: no black holes in the brain are benign. *Pract. Neurol.* 8 222–228. 10.1136/jnnp.2008.153601 18644908

[B30] O’Bryant S.E. Waring S.C. Cullum C.M. Hall J. Lacritz L. Massman P.J. (2008). Staging dementia using clinical dementia rating scale sum of boxes scores: a texas alzheimer’s research consortium study. *Arch. Neurol.* 65 1091–1095. 18695059 10.1001/archneur.65.8.1091 PMC3409562

[B31] Pantoni L. (2010). Cerebral small vessel disease: from pathogenesis and clinical characteristics to therapeutic challenges. *Lancet Neurol.* 9 689–701. 10.1016/s1474-4422(10)70104-6 20610345

[B32] Promjunyakul N. Lahna D. Kaye J.A. Dodge H.H. Erten-Lyons D. (2015). Characterizing the white matter hyperintensity penumbra with cerebral blood flow measures. *Neuroimage Clin.* 8 224–229. 10.1016/j.nicl.2015.04.012 26106546 PMC4473817

[B33] Promjunyakul N.O. Lahna D.L. Kaye J.A. Dodge H.H. Erten-Lyons D. Rooney W.D. (2016). Comparison of cerebral blood flow and structural penumbras in relation to white matter hyperintensities: a multi-modal magnetic resonance imaging study. *J. Cereb. Blood Flow Metab.* 36 1528–1536. 10.1177/0271678x16651268 27270266 PMC5010096

[B34] Rane S. Koh N. Boord P. Madhyastha T. Askren M.K. Jayadev S. (2018). Quantitative cerebrovascular pathology in a community-based cohort of older adults. *Neurobiol. Aging* 65 77–85. 10.1016/j.neurobiolaging.2018.01.006 29452984 PMC5871567

[B35] Rosenberg G.A. Wallin A. Wardlaw J.M. Markus H.S. Montaner J. Wolfson L. (2015). Consensus statement for diagnosis of subcortical small vessel disease. *J. Cereb. Blood Flow Metab.* 36 6–25. 10.1038/jcbfm.2015.172 26198175 PMC4758552

[B36] Schneider J.A. Arvanitakis Z. Bang W. Bennett D.A. (2007). Mixed brain pathologies account for most dementia cases in community-dwelling older persons. *Neurology* 69 2197–2204. 10.1212/01.wnl.0000271090.28148.24 17568013

[B37] Shi Y. Thrippleton M.J. Makin S.D. Marshall I. Geerlings M.I. de Craen A.J. (2016). Cerebral blood flow in small vessel disease: a systematic review and meta-analysis. *J. Cereb Blood Flow Metab.* 36 1653–1667. 10.1177/0271678x16662891 27496552 PMC5076792

[B38] Staals J. Booth T. Morris Z. Bastin M.E. Gow A.J. Corley J. (2015). Total MRI load of cerebral small vessel disease and cognitive ability in older people. *Neurobiol. Aging* 36 2806–2811. 10.1016/j.neurobiolaging.2015.06.024 26189091 PMC4706154

[B39] Staffaroni A.M. Asken B.M. Casaletto K.B. Fonseca C. You M. Rosen H.J. (2020a). Development and validation of the uniform data set (v3.0) executive function composite score (UDS3-EF). *Alzheimer’s Dementia* 2020:12214. 10.1002/alz.12214 33215852 PMC8044003

[B40] Staffaroni A.M. Bajorek L. Casaletto K.B. Cobigo Y. Goh S.Y.M. Wolf A. (2020b). Assessment of executive function declines in presymptomatic and mildly symptomatic familial frontotemporal dementia: NIH-EXAMINER as a potential clinical trial endpoint. *Alzheimer’s Dementia* 16 11–21. 10.1016/j.jalz.2019.01.012 PMC6842665 31914230

[B41] Staffaroni A.M. Cobigo Y. Elahi F.M. Casaletto K.B. Walters S.M. Wolf A. (2019). A longitudinal characterization of perfusion in the aging brain and associations with cognition and neural structure. *Hum Brain Mapp* 40 3522–3533. 31062904 10.1002/hbm.24613 PMC6693488

[B42] Sun Y. Cao W. Ding W. Wang Y. Han X. Zhou Y. (2016). Cerebral blood flow alterations as assessed by 3D ASL in cognitive impairment in patients with subcortical vascular cognitive impairment: a marker for disease severity. *Front. Aging Neurosci.* 8:211. 10.3389/fnagi.2016.00211 27630562 PMC5005930

[B43] Tatu L. Moulin T. Bogousslavsky J. Duvernoy H. (1998). Arterial territories of the human brain: cerebral hemispheres. *Neurology* 50 1699–1708. 10.1212/wnl.50.6.1699 9633714

[B44] US Census Bureau. (2016). *National Population Projections.* https://www.census.gov/data/tables/2017/demo/popproj/2017-summary-tables.html

[B45] Wallin A. Román G.C. Esiri M. Kettunen P. Svensson J. Paraskevas G.P. (2018). Update on vascular cognitive impairment associated with subcortical small-vessel disease2. *J. Alzheimers Dis.* 62 1417–1441. 10.3233/jad-170803 29562536 PMC5870030

[B46] Wang D.J.J. Alger J.R. Qiao J.X. Hao Q. Hou S. Fiaz R. (2012). The value of arterial spin-labeled perfusion imaging in acute ischemic stroke comparison with dynamic susceptibility contrast-enhanced MRI. *Stroke* 43 1018–U1200. 22328551 10.1161/STROKEAHA.111.631929 PMC3314714

[B47] Wardlaw J.M. Doubal F. Armitage P. Chappell F. Carpenter T. Munoz Maniega S. (2009). Lacunar stroke is associated with diffuse blood-brain barrier dysfunction. *Ann. Neurol.* 65 194–202. 10.1002/ana.21549 19260033

[B48] Yata K. Nishimura Y. Unekawa M. Tomita Y. Suzuki N. Tanaka T. (2014). In vivo imaging of the mouse neurovascular unit under chronic cerebral hypoperfusion. *Stroke* 45 3698–3703. 10.1161/strokeaha.114.005891 25370583

[B49] You S.C. Geschwind M.D. Sha S.J. Apple A. Satris G. Wood K.A. (2014). Executive functions in premanifest Huntington’s disease. *Mov. Disord.* 29 405–409. 10.1002/mds.25762 24375511 PMC4029327

[B50] Yushkevich P.A. Piven J. Hazlett H.C. Smith R.G. Ho S. Gee J.C. (2006). User-guided 3D active contour segmentation of anatomical structures: significantly improved efficiency and reliability. *Neuroimage* 31 1116–1128. 10.1016/j.neuroimage.2006.01.015 16545965

